# Electrical Contacts on Silicon Nanowires Produced by Metal-Assisted Etching: a Comparative Approach

**DOI:** 10.1186/s11671-016-1689-x

**Published:** 2016-10-20

**Authors:** L. D’Ortenzi, R. Monsù, E. Cara, M. Fretto, S. Kara, S. J. Rezvani, L. Boarino

**Affiliations:** 1 Nanoscience and Materials Division, INRiM (Istituto Nazionale di Ricerca Metrologica), Strada delle Cacce 91, 10135 Turin, Italy; 2 Politecnico di Torino, Corso Duca degli Abruzzi, 24, 10129 Turin, Italy; 3 Department of Chemical Engineering, Faculty of Technology, University of Blida 1, route de SOUMÂA B.P. 270, 09000 Blida, Algeria; 4 Centre de Recherche en Technologie des Semi-conducteurs pour l’Energétique (CRTSE), Thin Films, Surface and Interface Division, 02, Bd. Dr. Frantz Fanon, B.P. 140, Alger-7 Merveilles, 16038 Algiers, Algeria; 5 Università di Camerino, Via Madonna delle Carceri 9, 62032 Camerino, Italy

**Keywords:** Metal-assisted chemical etching, Nanosphere lithography, Silicon nanowires, Electrical contacts, Electron beam lithography, Focused ion beam, Schottky barrier, Rough surface

## Abstract

Silicon nanowires fabricated by metal-assisted chemical etching can present low porosity and a rough surface depending on the doping level of the original silicon wafer. In this case, wiring of silicon nanowires may represent a challenging task. We investigated two different approaches to realize the electrical contacts in order to enable electrical measurement on a rough silicon nanowire device: we compared FIB-assisted platinum deposition for the fabrication of electrical contact with EBL technique.

## Background

In the last decades, the study and the development of one-dimensional structures such as silicon nanowires (SiNWs) has produced a great interest in the scientific community. Because of their high aspect ratio and their electrical, thermal and optical properties, SiNWs are employed for a great variety of applications, such as electronics [1–3], chemical and biological sensing with high sensitivity [4], photovoltaic devices [5, 6], thermoelectric applications [7, 8] and rechargeable lithium-ion battery [9, 10].

The techniques used to synthesize nanowires are many, and among all, our study refers to the combination supramolecular self-assembly of polystyrene nanospheres and metal-assisted chemical etching (MACE) [11, 12], a technique based on electroless etching of silicon in the presence of a noble metal acting as a catalyst [13]. With this approach, it is possible to fabricate ordered wires with a well-defined diameter and spatial distribution. MACE is a cheap method which allows to achieve 3D morphologies strictly dependent on the surface metal patterning, thickness and distribution and guarantees a good control over the nanowires orientation with respect to the substrate [14]. Besides, MACE does not introduce any obvious crystallographic defects induced by the etching solution or any limitation to the wire size.

Depending on the initial substrate doping level, silicon nanowires present different percentage of porosity [15] which affects the basic mechanical and electrical properties of the nanostructure, influencing the depletion region, the band structure and charge traps as well as complicating the realization of proper electrical contacts [16]. A reliable and robust approach to fabricate electrical contacts on SiNWs is required in order to discriminate different contribution to measured electrical features arising from intrinsic properties of silicon nanowire or from spurious effects such as defective electrical contacts.

At the best of our knowledge, literature currently lacks a consistent analysis of electrical characteristics of silicon nanowires independently on their porosity content. In this regard, our research concerns the comparison of distinct techniques for the manufacture of single silicon nanowire devices, aiming to determine the most suitable method for the realization of a good ohmic contact and for electrical characterization at low temperature.

We chose to compare two methods; the first one includes electron beam lithography (EBL) followed by sputter-etching and Au-Pd deposition, while the second technique relies on the use of a focused ion beam (FIB) along with a gas injection system (GIS) for the deposition of platinum contacts. We performed I–V characterization on a single kind of silicon nanowire with greatly rough superficial features in order to highlight the advantages and drawbacks of the two contacting approaches.

## Methods

### Silicon Nanowire Fabrication

We use the metal-assisted chemical etching (MACE) process to fabricate porous silicon nanowires, starting from a boron-doped bulk silicon wafer, with an electrical resistivity of 8–12 mΩ cm and crystal orientation 〈100〉. When using this technique, a method to manage the diameter of the nanowires is the patterning of a noble metal film by means of a mask of self-assembled polystyrene nanospheres. The obtained patterning, commonly referred as “antidot” structure, sinks in the bulk silicon during MACE, and the remaining structures emerge as extrusion reporting the same diameter of the holes in the metallic layer.

The sequential processing steps are the following: after silicon wafer cleaning in acetone and ethanol, functionalization in a bath of piranha solution (H_2_SO_4 (96 %)_:H_2_O_2 (30 %)_ = 3:1) at 80 °C for 1 h making the surface hydrophilic. Then monodisperse colloidal aqueous solution of PS nanobeads (diameter of 260 nm) was spun on the Si substrate; in this way, a hexagonal close-packed monolayer of PS nanospheres was achieved on large area. Subsequently, we performed an etching in Ar^+^ plasma (RF power = 75 W and pressure = 10^−2^ mbar) to reduce the spheres diameter up to 90–110 nm, changing the monolayer in a hexagonal non-close-packed structure. In the next step, a 20-nm-thick gold film was deposited by electron beam evaporation onto the Si substrate masked with the nanosphere layer. After removing the spheres in ultrasonic bath with ethanol, the process resulted in a continuous metallic film with an array of defined circular voids. Finally, MACE was performed dipping the sample in a chemical solution composed of hydrofluoric acid, hydrogen peroxide and ethanol, with 30:1:20 ratio by volume, obtaining silicon nanowires with length ranging from 6 to 8 μm.

### Single Si Nanowire Device Fabrication

In a preparatory stage, we realized sub-millimetric circuits providing probe paths and pads for electrical measurement. We fabricated electrical circuits on a silicon substrate with a 500-nm-thick layer of silicon dioxide as insulation layer. A resist layer, spun on the substrate, was patterned using photolithography with a customized optical mask. Subsequently, we performed a sputter deposition of 200-nm-thick film of niobium, obtaining the probe circuit after lift-off in acetone. We transferred SiNWs from the original substrate to a square area delimited by the ends of Nb paths of the probe circuit by using a thin copper wire (diameter = 0.1 mm) as scratch tool. The wires were randomly distributed in tangles on the new substrate; thus, we accurately selected few isolated SiNWs by means of a dual beam Quanta 3D Microscope (FEI) and stuck them on the substrate by depositing platinum spots with a gas injection system (GIS). The excess of SiNWs was then removed with an ultrasonic bath in ethanol. At this step, two different methods of wiring were adopted in order to connect single nanowires to the Nb paths. In the first one, we used a customized electron beam ithography (EBL) process; firstly, we spun a PMMA layer on the substrate and then we exposed the resist to the e-beam of Quanta 3D Microscope, thus patterning sub-micrometric paths to match the probe circuit and the nanowires. Finally, electrical contacts were realized with a sputter deposition of Au_32_-Pd_68_ alloy preceded by a sputter-etching process with Ar^+^. In the second method, the wiring was achieved directly in the Quanta 3D Microscope chamber. In fact, we used the focused ion beam in conjunction with GIS and a patterning software tool, which allows a spatial-controlled deposition of platinum; we realized Pt paths connecting SiNWs to the probe circuit in a single-step process.

### Electrical Measurement

The single SiNW devices were electrically characterized at various temperature ranging from 100 to 300 K; the measurements were performed in a thermally controlled environment (Janis ST-100H cryostat) consisting of a cold finger column inserted in a vacuum chamber. The samples were mounted on the holder at the end of the cold finger, and a thin Kapton film (12.5 μm) was placed between the samples and the holder to ensure electrical insulation of the circuit while providing a good thermal contact. The vacuum chamber was connected to a scroll pump (Varian SH-100) and to a turbo-molecular pump (Alcatel ATP 80), leading to a residual pressure around 10^−5^ mbar. The value of temperature was acquired with a platinum Pt-100 resistor embedded into the cold finger, while a controller (Lake-Shore 331), coupled with a heater and a liquid nitrogen transfer-line, tuned the temperature to the set-point value. Kapton-covered cables inside the chamber provided electrical wiring of the samples to external coaxial plugs. We used a Keithley 6430 Sub-Femtoamp SourceMeter with remote preamplifier for electrical measurement; external noises were minimized using a short triaxial cable connected to the coaxial plugs of the cryostat through a specific adapter. The SourceMeter system provides three signal terminals, namely Hi and Low terminals for the electrical measurement and the Guard terminal for the guard ring circuit. The two main signals were connected to the probe pads of the single SiNW devices, while the guard terminal was bonded to the backside of the samples to avoid any current leakage in the substrate.

## Results and Discussions

**Fig. 1:**
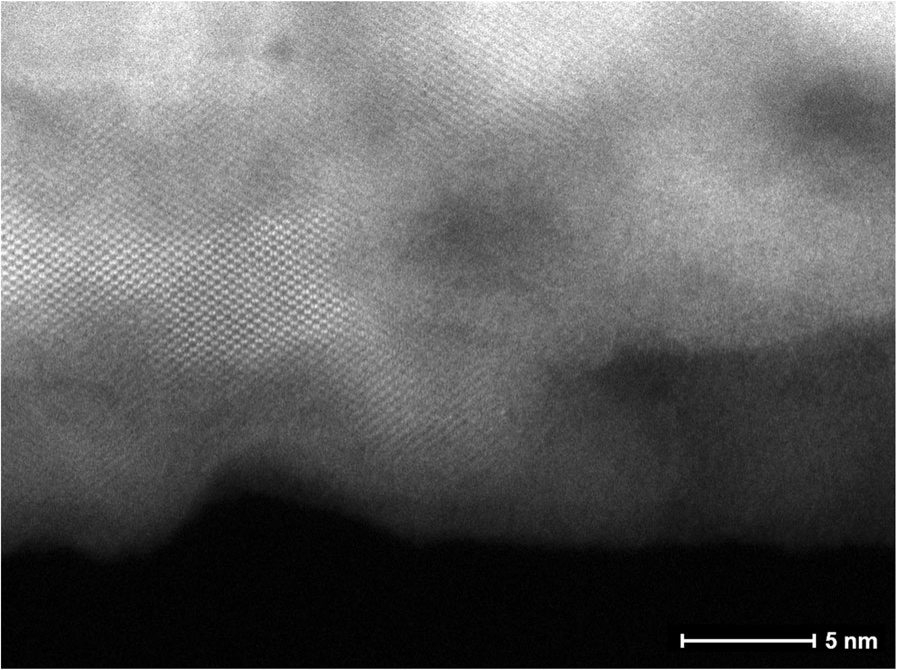
TEM image of a silicon nanowire fabricated by means of metal-assisted chemical etching with HF:H2O2:EtOH = 30:1:20 etching solution. The original resistivity of silicon was 8–12 mΩ cm. The sample presents a high surface roughness; however, no quantitative considerations on the porosity content can be done, due to the intrinsic limitation of TEM analysis, since the transmission signal is relative to the whole volume of the wire. On the other hand, several crystalline domains are preserved inside the nanowire

SiNWs obtained with the described MACE process presented high surface roughness. Although a crystalline order was revealed with a transmission electron microscope (TEM) analysis (Fig. 1), the presence of pores in the nanowire structure cannot be excluded.

Indeed, the picture produced by the transmitted signal is a projection of the whole depth of the material crossed by the electrons; for this reason, it is poorly suitable for detection of empty pores. Other studies [17] confirm a low level of porosity in Si nanowires fabricated in the same conditions.

We first characterized SiNWs wired with FIB/GIS technique. It is known that the decomposition of the organic-metallic gas released by GIS induces on the wire contamination by oxygen, carbon and gallium [18–20], in the area surrounding the platinum paths. This surface contamination can provide alternative current pathways during electrical characterization, falsifying the real electrical properties of the nanowire. In the early measurement, we observed in fact a very high current (around 400 μA at a voltage of 5 V), which appeared to be quite unlikely even for a high-doped nanowire. Indeed, in our case the SiNWs exhibited a resistivity of 12–13 mΩ cm. Even though this result is comparable with the bulk resistivity, it seems to be too high for a SiNWs with a high surface roughness. We first faced this issue digging a trench with the FIB in order to interrupt the current crossing between the Pt paths. The related electrical measurements turned out very similar, with just a slight decrease of the current after the FIB milling. The lack of efficiency of this method suggested us to deeper investigate the role of contamination in the electrical contact. In order to understand the spatial distribution of it, we observed the samples with SEM at low acceleration voltage (0.5–1 kV) to obtain a higher contribution from the surface to the SEM signal. In this manner, we discovered two kinds of contamination. The first one is due to the imaging with the ionic beam. The second one is related to the Pt deposition itself.

**Fig. 2:**
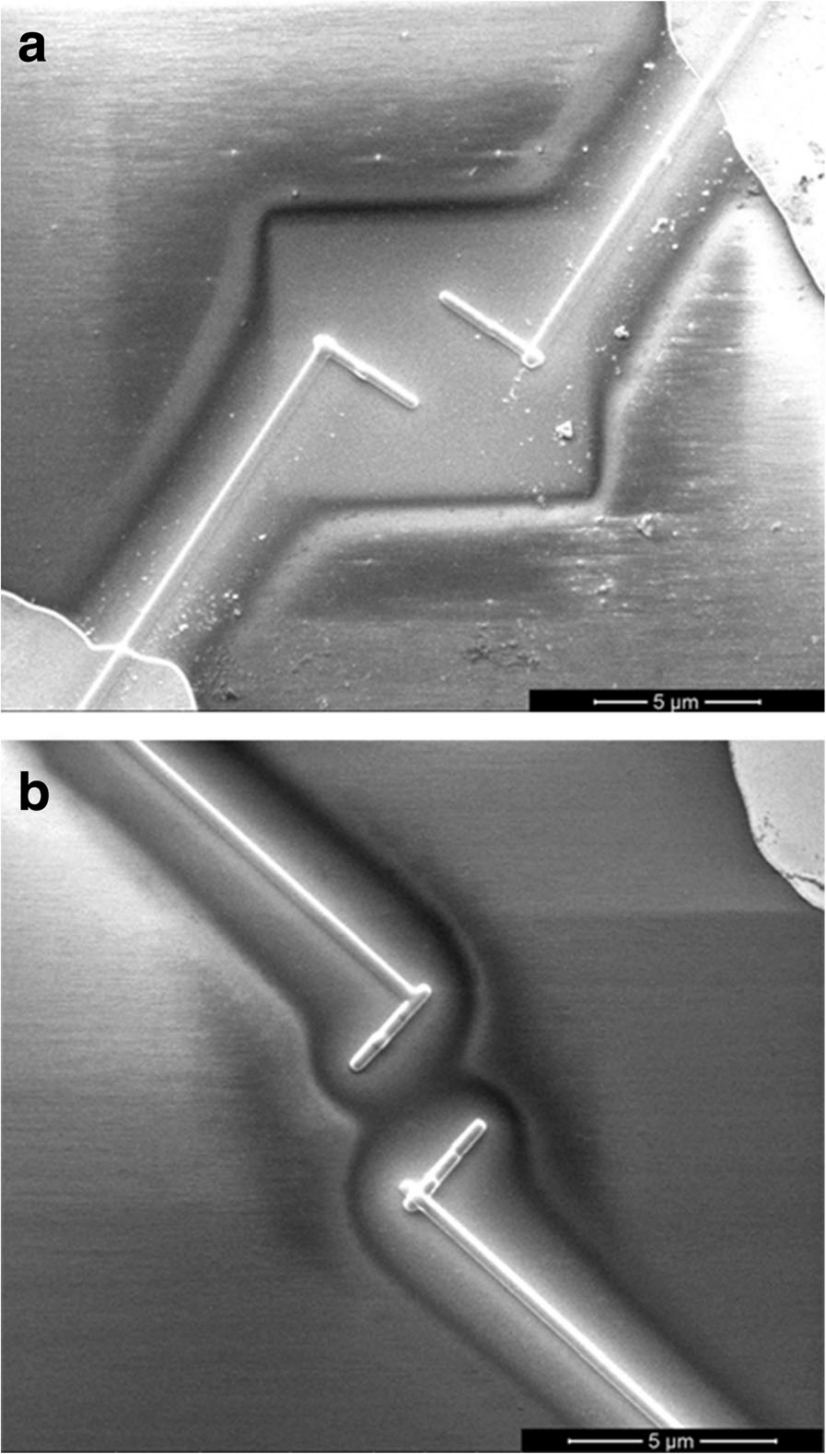
SEM images taken at 1 kV of acceleration voltage of fake devices consisting in couples of Pt electrodes. This e-beam setting allows us to highlight the surface contamination. The relief appearance is due to the electrical charging of the insulating surface. This effect also makes the scanning lines of the e-beam visible. In the areas where the lines are not visible, the surface results to be conductive. **a** A square on the device longer exposed to the FIB is evident, denoting a strong contamination in that region. On the other hand, in **b**, representing the short exposed device, any structure due to the FIB exposure is detectable. In both cases a halo around the Pt electrodes is observable

**Fig. 3:**
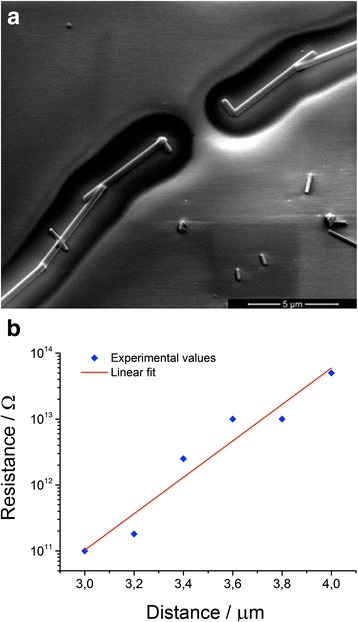
**a** SEM images taken at 1 kV of acceleration voltage of a couple of Pt electrodes at 4.0 μm of distance, made with a layout that minimizes the overlapping area. The relief appearance is explained in the capture of Fig. 2. At this distance, the halos around the Pt electrodes appear to be by around 1 μm separated. In **b**, a semi-log graph shows the measured resistance between the electrodes as a function of the distance. The linear fit points out an exponential law

Observing silicon nanowires with ionic beam is required for aligning and patterning Pt electrodes. However, even a moderate exposure to the ionic beam can dramatically contaminate the surface. To reproduce what happens during this process, we fabricated two fake devices composed of only a couple of Pt electrodes placed 3.0 μm away from each other, with no SiNW contacted. Previously to the Pt deposition, the surface of one device was exposed for 20 s to a continuous image acquisition made with a 10-pA FIB current at 25K magnification, while the other one was exposed only to a single frame acquisition with a scanning speed of 100 μs at the same image condition, limiting the dose to the lower value possible for the device fabrication. Figure 3 shows what we observed by SEM with 1 kV of acceleration voltage. The device longer exposed to the FIB exhibits a glaring square surrounding the Pt electrodes (Fig. 2a), whereas any structure is observable in the briefly exposed one (Fig. 2b).

The related electrical resistance between the Pt electrodes was 36 kΩ and 35 GΩ, respectively, revealing a deep influence of the FIB exposure contamination, which reduces the resistance of about a six-order of magnitudes in this case. Another fact that arises from the Fig. 2 is the halo around the Pt electrodes. This contamination has a width of approximately 1.5 μm in each side of the paths. Using the FIB in the single frame image acquisition mode to minimize the exposure, we fabricated couples of Pt electrodes with no wire crosswise at several distances from 3.0 to 4.0 μm. We also adopted a different layout in order to reduce the overlapping area between the paths (Fig. 3a). The resulting resistance shows an exponential behaviour with the distance (Fig. 3b), going from 1 × 10^11^ Ω at 3.0 μm to 5 × 10^13^ Ω at 4.0 μm. It is clear that the device can be thought as on open circuit at 4.0 μm.

**Fig. 4:**
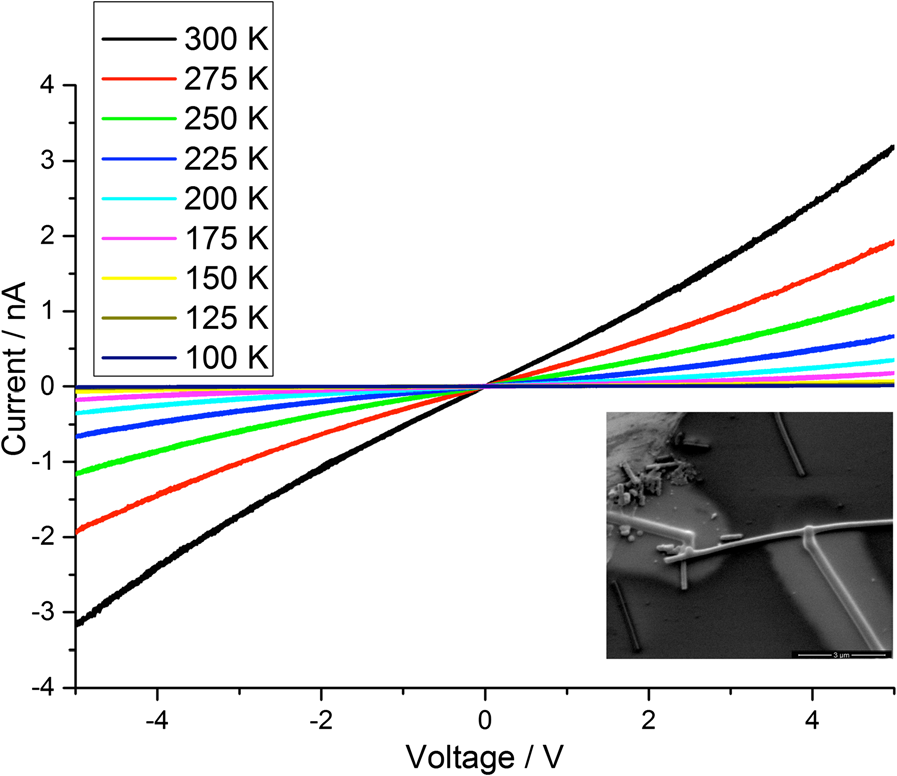
I–V curves of SiNWs contacted with the FIB/GIS method at different temperature (from 100 to 300 K). Each *curve* represents two voltamperogrames cycles. In the *inset*, a SEM image of the measured SiNW taken at 500 V of acceleration voltage. The halo is well visible

Taking in account these results, we contacted SiNWs with FIB/GIS technique adopting a distance of 4.0 μm between the electrodes and using the single frame image acquisition mode for the FIB. Typical electrical characteristics as a function of temperature are shown in Fig. 4. Each curve represents two voltamperogrames cycles. The characteristics appear to be quite linear in the considered range of bias voltage, as well as stable, reproducible and with no hysteresis.

**Fig. 5:**
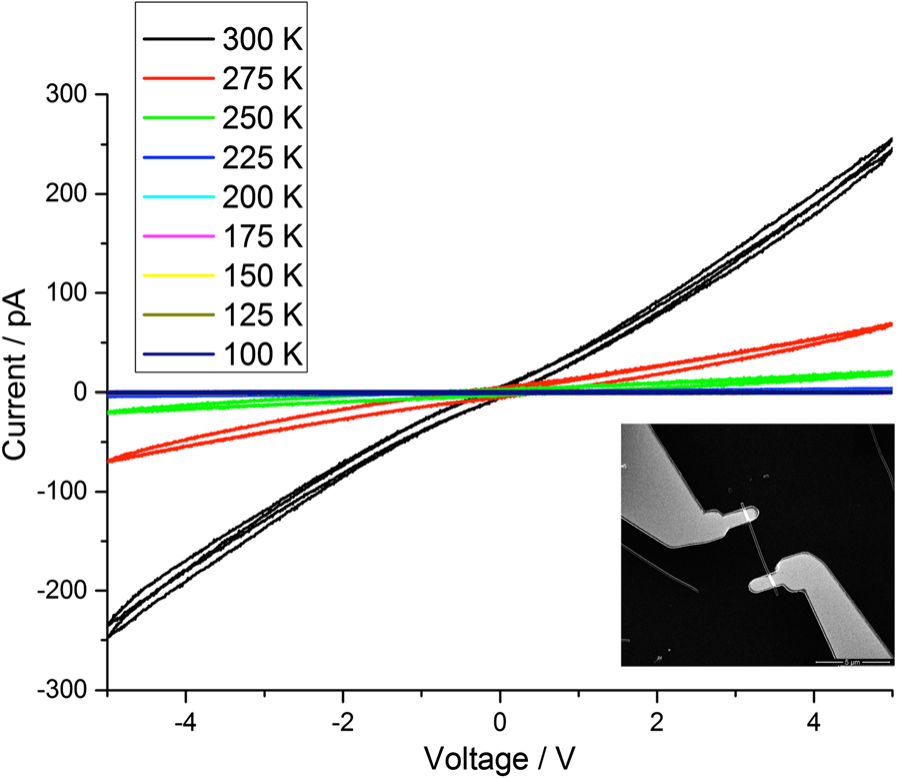
I–V curves of SiNWs contacted with the EBL method at different temperature (from 100 to 300 K). Each *curve* represents two voltamperogrames cycles. A little hysteresis is detectable as well as a slight instability of the curves. In the *inset*, a SEM image of the measured SiNW

EBL method does not involve surface contamination issues. Using the procedure described in the Methods section, we wired SiNW patterning electrodes at the same distance (4.0 μm) of the previous case. In Fig. 5, electrical characteristics of SiNW EBL wired are reported.

Even in this case, each curve represents two voltamperogrames cycles. A little hysteresis is also detectable as well as a slight instability of the curves, and the measured currents are one order of magnitude smaller than the FIB/GIB wired case. This finding could be due to the halo around the Pt paths in the FIB/GIS method, which reduces the effective length of the SiNW contacted. Taking in account our study, we can estimate this length to around 1 μm, which is the uncontaminated segment of the nanowire. Hysteretical characteristics have been commonly observed in SiNWs, due to the presence of surface and interface charge-trapping states on the nanowire surface [21, 22].

**Fig. 6:**
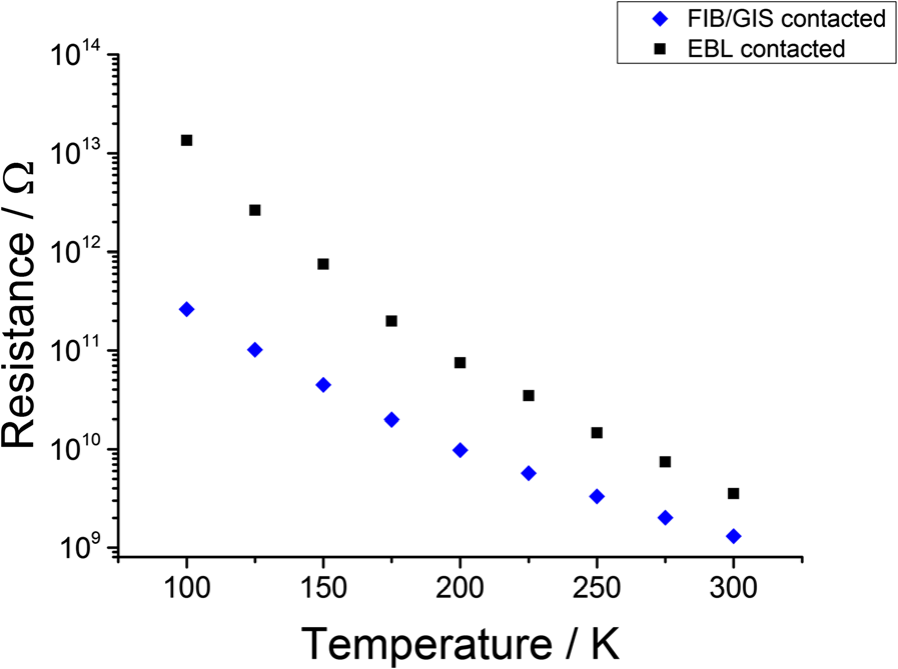
Resistances of 4.0-μm long SiNWs wired with FIB/GIS method compared with a 1.0-m long SiNW wired with the EBL method as a function of the temperature. The resistances are calculated by means of a linear fit in the voltage range of 4.8‒5.0 V. The small difference in resistance observable at higher temperature could be attributed to a discrepancy on the effective lengths. However, at lower temperature, this difference becomes bigger up to one order of magnitude. We believe this finding could be the sign of a greater Schottky barrier in the EBL wired SiNWs

For this reason, in Fig. 6, we compared the resistances of a 4-μm long FIB/GIS wired SiNW with a 1-μm long EBL wired one. The resistances as a function of the temperature are evaluated by means of a linear fit in the voltage range of 4.8–5.0 V.

The values of the resistance at higher temperature are very similar. The slight discrepancy could be attributed to a little mismatch of the effective lengths. However, going down with the temperature, this difference becomes bigger up to one order of magnitude at 100 K.

A plausible explanation of the observed behaviour could be the presence of a Schottky barrier at the metal/SiNW interface in the EBL method. Many metals can arise this effect when semiconducting nanowires are contacted [23]. Especially in low-dimensional devices, where very high electric fields can occur, the current can pass through the barrier by the tunnelling effect [24]. However, at low temperature, the probability of a tunnel event becomes smaller, limiting the current.

On the other hand, studies in literature [25, 26] demonstrate that the action of Ga+ ions, during the Pt deposition in the FIB/GIS method, can induce amorphization in the crystalline silicon. They claim that a Fermi-level pinning occurs due to the localized states in the disordered structures and is responsible for a severe lowering of the Schottky barrier.

These considerations could explain the high discrepancy of the resistances at low temperature. In both cases, the SiNW resistivity is greater than the bulk one by some orders of magnitude.

## Conclusions

In summary, we have examined two different methods for contacting nanowires widely used in the nanotechnology field. The roughness of SiNWs obtained via MACE hinders the wiring process. Our comparative study allows to underline some relevant feature of the two techniques. FIB/GIS method is efficient in terms of electrical contact on the silicon nanowires even if it requires lots of effort for limiting the contamination of the surface. We have found a partial solution using nanowires longer than 4.0 μm and restricting the exposure to the ion beam as low as possible. EBL technique is much more clean but does not eliminate the Schottky barrier between the electrode and the nanowire. Nevertheless, this method introduces some chemical steps that could alter the electrical properties of the silicon nanowires, especially if their surface contains pores.

Further studies are needed for solving the contamination issue in the FIB/GIS method and better comparing the two techniques.

## Abbreviations

FIB: Focused ion beam
GIS: Gas injection system
PMMA: Polymethyl methacrylate
PS: Polystyrene
SEM: Scanning electron microscope
SiNWs: Silicon nanowires
TEM: Transmission electron microscope
